# Supplementary material for: Single-nucleus multiomics reveals the gene regulatory networks underlying sex determination of murine primordial germ cells
Source: eLife. 2025 Mar 10;13:RP96591. doi: 10.7554/eLife.96591 (PMC11893106; doi:10.7554/eLife.96591)
Supplement: Supplementary file 1. [file elife-96591-supp1.docx]

**Supplementary File 1. Sequencing and QC statistics for single-nucleus multiome libraries of E11.5-E13.5 XX and XY gonads.**

| **Sample** | **Pooled Paired Gonads (Number)** | **Raw Reads** | **Barcode** | **Mapped** | **Saturation** | **Cells** | **Reads/cell** | **Median UMI/cell** | **Median genes/cell** |
| --- | --- | --- | --- | --- | --- | --- | --- | --- | --- |
| **E11.5 F_R1** | 19 | 448,115,792 | 93% | 91% | 38% | 20,000 | 22,406 | 3,665 | 2,030 |
| **E11.5 F_R2** | 11 | 812,813,546 | 94% | 92% | 68% | 10,008 | 81,216 | 8,184 | 2,798 |
| **E11.5 M_R1** | 18 | 193,672,880 | 93% | 94% | 35% | 8,080 | 23,969 | 2,790 | 1,708 |
| **E11.5 M_R2** | 12 | 719,219,983 | 94% | 91% | 64% | 4,960 | 145,326 | 5,934 | 3,512 |
| **E12.5 F_R1** | 12 | 498,266,895 | 93% | 94% | 43% | 18,696 | 26,651 | 4,620 | 2,422 |
| **E12.5 F_R2** | 11 | 654,449,771 | 94% | 91% | 66% | 4,949 | 131,946 | 8,351 | 3,504 |
| **E12.5 M_R1** | 8 | 425,422,729 | 93% | 91% | 44% | 14,897 | 28,558 | 5,268 | 2,682 |
| **E12.5 M_R2** | 10 | 951,070,062 | 94% | 92% | 74% | 5,864 | 162,188 | 9,194 | 3,446 |
| **E13.5 F_R1** | 3 | 135,990,358 | 95% | 92% | 86% | 1,555 | 83,036 | 3,608 | 2,121 |
| **E13.5 F_R2** | 5 | 398,709,188 | 95% | 92% | 73% | 6,369 | 74,137 | 6,013 | 2,831 |
| **E13.5 M_R1** | 3 | 129,120,442 | 94% | 94% | 82% | 2,040 | 66,662 | 5,132 | 2,642 |
| **E13.5 M_R2** | 7 | 472,180,945 | 94% | 91% | 71% | 6,597 | 60,438 | 6,546 | 2,969 |
